# In Silico Evaluation of Iranian Medicinal Plant Phytoconstituents as Inhibitors against Main Protease and the Receptor-Binding Domain of SARS-CoV-2

**DOI:** 10.3390/molecules26185724

**Published:** 2021-09-21

**Authors:** Seyyed Sasan Mousavi, Akbar Karami, Tahereh Movahhed Haghighi, Sefren Geiner Tumilaar, Fatimawali, Rinaldi Idroes, Shafi Mahmud, Ismail Celik, Duygu Ağagündüz, Trina Ekawati Tallei, Talha Bin Emran, Raffaele Capasso

**Affiliations:** 1 Department of Horticultural Science, School of Agriculture, Shiraz University, Shiraz 71441, Iran; seyedsasanmousavi66@gmail.com (S.S.M.); akbarkarami@shirazu.ac.ir (A.K.); tmovahhed@gmail.com (T.M.H.); 2 Pharmacy Study Program, Faculty of Mathematics and Natural Sciences, Sam Ratulangi University, Manado 95115, Indonesia; sefrentumilaar1@gmail.com (S.G.T.); fatimawali@unsrat.ac.id (F.); 3 The University Center of Excellence for Biotechnology and Conservation of Wallacea, Sam Ratulangi University, Manado 95115, Indonesia; 4 Department of Pharmacy, Faculty of Mathematics and Natural Sciences, Universitas Syiah Kuala, Kopelma Darussalam, Banda Aceh 23111, Indonesia; rinaldi.idroes@unsyiah.ac.id; 5 Microbiology Laboratory, Department of Genetic Engineering and Biotechnology, University of Rajshahi, Rajshahi 6205, Bangladesh; shafimahmudfz@gmail.com; 6 Department of Pharmaceutical Chemistry, Faculty of Pharmacy, Erciyes University, Kayseri 38039, Turkey; ismailcelik@erciyes.edu.tr; 7 Department of Nutrition and Dietetics, Faculty of Health Sciences, Gazi University, Emek, Ankara 06490, Turkey; duyguturkozu@gazi.edu.tr; 8 Department of Biology, Faculty of Mathematics and Natural Sciences, Sam Ratulangi University, Manado 95115, Indonesia; 9 Department of Pharmacy, BGC Trust University Bangladesh, Chittagong 4381, Bangladesh; 10 Department of Agricultural Sciences, University of Naples Federico II, Portici, 80055 Naples, Italy

**Keywords:** medicinal plant, coronavirus, main protease, receptor-binding domain, molecular dynamics simulation, molecular docking, inhibitor, chelidimerine

## Abstract

The novel coronavirus disease 2019 (COVID-19) is caused by the severe acute respiratory syndrome coronavirus 2 (SARS-CoV-2), which initially appeared in Wuhan, China, in December 2019. Elderly individuals and those with comorbid conditions may be more vulnerable to this disease. Consequently, several research laboratories continue to focus on developing drugs to treat this infection because this disease has developed into a global pandemic with an extremely limited number of specific treatments available. Natural herbal remedies have long been used to treat illnesses in a variety of cultures. Modern medicine has achieved success due to the effectiveness of traditional medicines, which are derived from medicinal plants. The objective of this study was to determine whether components of natural origin from Iranian medicinal plants have an antiviral effect that can prevent humans from this coronavirus infection using the most reliable molecular docking method; in our case, we focused on the main protease (M^pro^) and a receptor-binding domain (RBD). The results of molecular docking showed that among 169 molecules of natural origin from common Iranian medicinal plants, 20 molecules (chelidimerine, rutin, fumariline, catechin gallate, adlumidine, astragalin, somniferine, etc.) can be proposed as inhibitors against this coronavirus based on the binding free energy and type of interactions between these molecules and the studied proteins. Moreover, a molecular dynamics simulation study revealed that the chelidimerine–M^pro^ and somniferine–RBD complexes were stable for up to 50 ns below 0.5 nm. Our results provide valuable insights into this mechanism, which sheds light on future structure-based designs of high-potency inhibitors for SARS-CoV-2.

## 1. Introduction

The novel coronavirus disease 2019 (COVID-19) has been declared a global pandemic and has resulted in millions of deaths worldwide. The disease appeared in late December 2019 in Wuhan, China, as a result of zoonotic transmission [1]. The virus that causes COVID-19, severe acute respiratory syndrome-related coronavirus 2 (SARS-CoV-2), was shown to share 96% genomic identity with the related bat coronavirus [2]. Coronaviruses belong to the family Coronaviridae, which is a family of enveloped single-stranded positive-sense RNA viruses. Therapeutic approaches such as neuraminidase inhibitors, nucleoside analogs, anti-inflammatory drugs, and antiviral drugs (e.g., remdesivir/lopinavir/ritonavir/nelfinavir/umifenovir/tenofovir disoproxil fumarate) have been suggested to treat COVID-19 [3,4,5,6,7,8]. Currently, more than 200 clinical trials analyzing these and other drugs have been registered on clinicaltrials.gov. Nevertheless, the clinical usefulness of remdesivir against COVID-19 infection remains unclear [9].

Traditional herbal medicines have been used since the early days of the COVID-19 outbreak. Notably, some of these traditional medicines were shown to result in the recovery of 90% of 214 patients treated in China [10]. Furthermore, some traditional herbal medicines served as inflammatory drugs for SARS-CoV-2 infection in healthy individuals and improved the health state of patients with mild or severe symptoms [11]. Several methods using medicinal plants have been recommended for the prevention of COVID-19. Moreover, to treat the disease, experts have recommended the use of various herbal mixtures according to the disease stage [12].

To fight COVID-19, various traditional antiviral medicines have been prescribed to infected patients with mild to moderate symptoms and have resulted in unexpected success in controlling the disease. However, the molecular mechanisms of how these herbal medicines interact with SARS-CoV-2 and how this virus causes COVID-19 have remained elusive. Some preliminary studies have investigated potential combinations that include the protease inhibitor lopinavir/ritonavir, which is commonly used to treat human immunodeficiency virus (HIV)/acquired immunodeficiency syndrome patients, for the treatment of COVID-19-infected patients [13,14]. In the present study, we investigated 169 components from 82 Iranian medicinal plants as potential candidates for COVID-19 management using in silico methods (molecular docking and molecular dynamics (MD) simulation). We explored the underlying molecular mechanisms of the computationally determined top candidates, which are key components in many traditional antiviral medicines for inhibiting the viral targets main protease (M^pro^) and receptor-binding domain (RBD). M^pro^ is an ideal antiviral target because it is involved in the processing of the corona-virus-encoded polyprotein that mediates the assembly of the transcription–replication machinery [15,16]. RBD exhibits a high affinity for the human angiotensin-converting enzyme 2 (hACE2) protein, which serves as the receptor for SARS-CoV-2 virus entry [17]. Therefore, if a small molecule can bind to RBD, it will hypothetically inhibit their viral infection of host cells. Several in vitro analyses have proven that RBD can be a target for viral entry inhibition [18,19]. The findings of the present study will provide researchers with opportunities to identify the correct drug(s) to combat COVID-19.

## 2. Results

### 2.1. Molecular Docking Analysis

In the present study, 82 plants were chosen from top Iranian medicinal plants, and a total of 169 components were docked (Table S1). Table S2 presents the binding affinity of the studied natural compounds to the receptors 6LU7 (M^pro^) and 6YLA (RBD) using AutoDock Vina. The docking process was repeated using AutoDock Tools, specifically for the best interaction generated by AutoDock Vina to ensure accuracy. Table 1 summarizes the findings. Lopinavir was used as a control for docking with M^pro^. This was based on prior research indicating that lopinavir was one of the first antiviral drugs used in clinical trials to treat COVID-19 where M^pro^ is the target of the antiviral drug [15]. The docking results indicated that there were no significant differences in the binding free energy values generated by AutoDock Vina and AutoDock Tools.

For M^pro^, 11 compounds (chelidimerine, catechin gallate, badrakemin acetate, withanolide G, samarcandin, somniferine, adlumidine, withanone, pelargonidin 3-glucoside, astragalin, and fumariline) achieved the best docking results based on binding energy. Moreover, the 12 compounds with the best docking results based on their binding energy for the RBD were withanolide G, chelidimerine, badrakemin acetate, withanone, samarcandin, pinoresinol 4-O-b-d-glucopyranoside, norsanguinarine, sanguinarine, adlumidine, pelargonidin-3 glucoside, somniferine, and fumariline. Table 2 (for M^pro^) and Table 3 (for RBD) show the interactions between amino acid residues on the receptors and the compounds. Lopinavir showed pi-sulfur bonds at M^pro^ with Cys:A145 and carbon H-bonds at His:A41. In addition, this drug showed the presence of conventional H-bonds on the amino acids Glu:A186, Gln:A189, Asn:A142, and Gly:A143 at M^pro^. Meanwhile, chelidimerine showed a pi-pi T-shaped bond at M^pro^ with His:A41 and a pi-donor H-bond at Cys:A145. H-bonds were formed between this compound and the amino acids Arg:A188, Thr:A190, Leu:A167, Gln:A189, Glu:A166, His:A164, Leu:A141, His:A163, Ser:A144, and Gly:A143.

**Figure 1:**
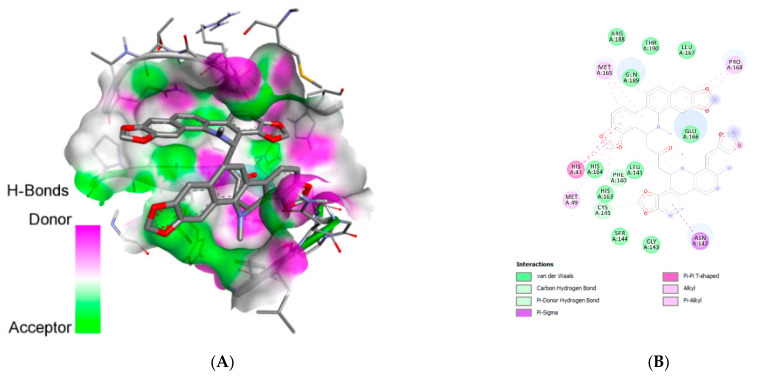
Three-dimensional (**A**) and two-dimensional (**B**) diagrams depicting the interaction of chelidimerine with the amino acid residues of the main protease (6LU7).

**Figure 2:**
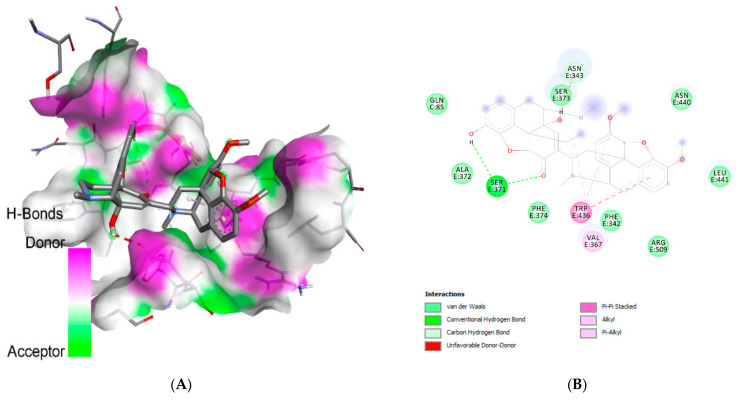
Three-dimensional (**A**) and two-dimensional (**B**) diagrams depicting the interaction of somniferine with the amino acid residues of the receptor-binding domain (6YLA).

At RBD, somniferine was observed to form a conventional H-bond with Ser:E:371; van der Waals interactions with Gln:C85; Ser:E373, Asn:E440, Leu:E441, Ala:E372, Phe:E374, Phe:E342, and Arg:E509; a pi-pi stacked with Trp:E436; and an alkyl bond with Val:E376. The 3D and 2D diagrams in Figure 1 illustrate the interactions between the receptor M^pro^’s amino acid residues and chelidimerine, while Figure 2 illustrates the interactions between the receptor-binding domain’s amino acid residues and somniferine. The selection of chelidimerine and somniferine was based on the results of docking, both of which showed good binding affinity for M^pro^ and RBD, respectively.

### 2.2. ADMET Analysis

The absorption, distribution, metabolism, excretion, and toxicity of chemicals (ADMET) all play critical roles in the discovery and development of new drugs. Therefore, pharmacokinetic and toxicity properties of the evaluated compounds that showed the best interaction with M^pro^ and RBD were also examined to provide confidence in the proficiency and safety of these compounds (Table 4). The average molecular weight of the selected compounds was below 500 g/mol, except for chelidimerine (720.7 g/mol), somniferine (608.7 g/mol), and cyanidin 3-O-rutinoside (595.5 g/mol). Additionally, several other parameters were evaluated, including carcinogenicity, hepatotoxicity, CNS permeability, CYP inhibition, and acute oral toxicity. The level of toxicity can be described as follows: class I (fatal if swallowed, LD50 ≤5 mg/kg), class II (fatal if swallowed, LD50 5 < LD50 ≤ 50 mg/kg), class III (toxic if swallowed, LD50 50 < LD50 ≤ 300 mg/kg), class IV (harmful if swallowed, LD50 300 < LD50 ≤ 2000 mg/kg), class V (maybe harmful if swallowed, LD50 2000 < LD50 ≤ 5000 mg/kg), class VI (nontoxic, LD50 > 5000 mg/kg).

### 2.3. In Silico Inhibition Constant

The predicted half-maximal inhibitory concentration (IC_50_) value was also evaluated (Table 5) to gain a better understanding of the plausible experimental antiviral activity of the studied compounds. The IC_50_ is a strong parameter for assessing the compound’s ability to halt biological processes by half and is widely used to represent the inhibitory impact of the compound [20]. The IC_50_ for M^pro^ was predicted to be 136.22 M, 25.90 nM and 776.89 nM for lopinovir, chelidimerine, and somniferine, respectively. Meanwhile, the IC_50_ values were 843.38 nM and 12.30 M for chelidimerine and somniferine, respectively, for RBD.

### 2.4. Molecular Dynamics Simulation Study

**Figure 3:**
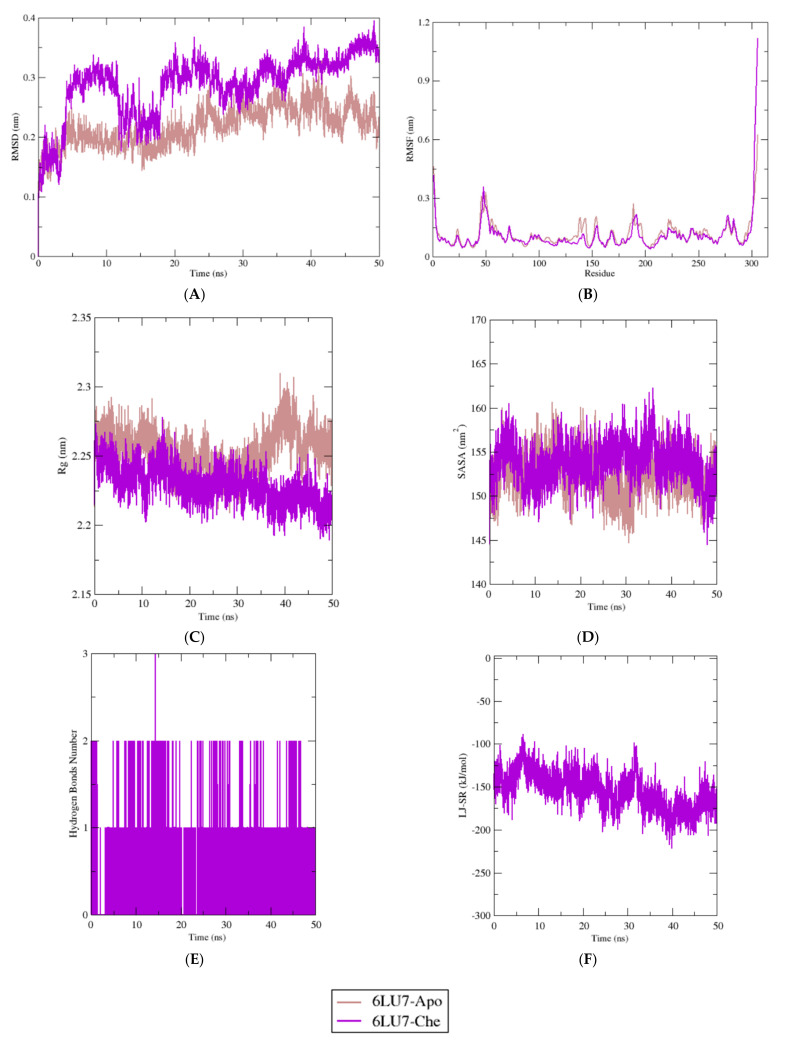
(**A**) Root-mean-square deviation (RMSD), (**B**) root-mean-square fluctuation (RMSF), (**C**) radius of gyration (Rg), (**D**) solvent accessible surface area (SASA), (**E**) intermolecular hydrogen bonds number, (**F**) short-range Lennard-Jones protein-ligand interaction energy analysis of the apo form (6LU7-Apo) and chelidimerine (6LU7-Che) holo form of SARS-CoV-2 M^pro^ throughout 50 ns.

**Figure 4:**
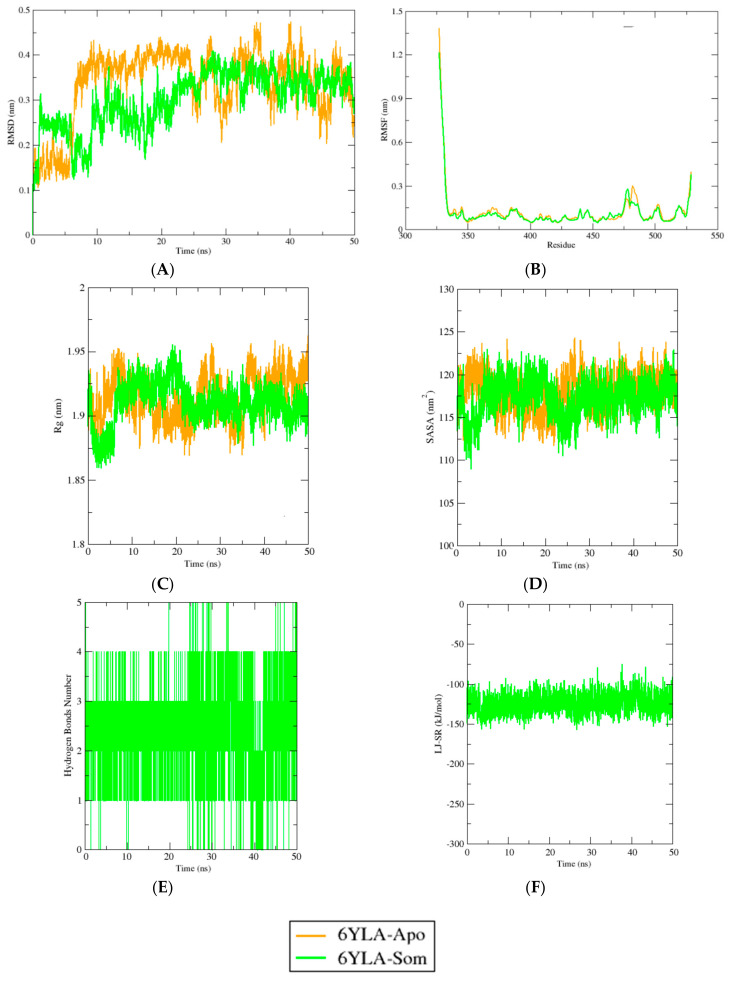
(**A**) RMSD, (**B**) RMSF, (**C**) Rg, (**D**) SASA, (**E**) intermolecular hydrogen bonds number, and (**F**) short-range Lennard-Jones protein-ligand interaction energy analysis of the apo form (6YLA-Apo), and somniferine (6YLA-Som) holo form of RBD of SARS-CoV-2 spike protein for 50 ns.

Chelidimerine had a good interaction with M^pro^, whereas somniferine showed good interaction with RBD. As a result, the analysis of the interactions of these two compounds with M^pro^ and RBD was continued using molecular dynamics simulations, as illustrated in Figure 3 and Figure 4. The study of MDS was processed for a timescale of 50 ns to analyze the stability and behavioral dynamics of the interaction between the ligand and the receptor in an aqueous environment. As shown in Figure 3, the RMSD profile for chelidimerine and protein complex was stable after 20 to 50 ns. The RMSF values were below 1.2 nm during the simulation process. Compared to chelidimerine and protein complex, the Rg values of the apo complex were more flexible, whereas those of the chelidimerine and protein complex were more contractile. The SASA profiles for both apo and the complex were similar during the simulation process. As can be seen in Figure 4, the RMSD value of the somniferine and RBD complex had a higher deviation during 5 to 20 ns but then maintained the stabilization of the complex. The RMSF values showed low fluctuations. The Rg profile of somniferine and RBD complex were stable after 20 ns. The SASA profiles of both systems fluctuated during the simulation, but they eventually stabilized at the end of the simulation.

## 3. Discussion

Molecular docking is one of the most popular methods in the field of computer-aided drug design (CADD) for the identification of new drug leads [21]. CADD is currently being used to rapidly annotate and analyze large drug libraries, thereby saving an immense amount of energy, time, and costs [22,23]. The current study examined a total of 169 compounds, as shown in Table S1. The docking results for AutoDock Vina and AutoDock Tools are shown in Table S2 and Table 1, respectively. The binding free energy values of the two tools were comparable, indicating that the docking results were valid. Both platforms are widely used for docking proteins and ligands. In general, the results indicated that Vina and AutoDock performed similarly well at discriminating between actives and decoys [24]. There are differences in binding free energy between each ligand and receptor, which means that not all ligands have the ability to interact well with receptors. From a biological and pharmacological perspective, these molecules, which are proposed as inhibitors of M^pro^ and RBD, have significant antiviral power according to bibliographical research and prior experiments. Numerous studies have identified M^pro^ and RBD as possible small molecule targets in the search for COVID-19 drugs [25,26,27,28].

The docking scores for the molecules that bind with the M^pro^ and RBD receptors are shown in Table 1. Chelidimerine (binding free energy to M^pro^ equaled −10.2 kcal/mol), an isoquinoline alkaloid, is an important compound in *Fumaria* species that can suppress the hepatitis B virus [29]. Rutin is another candidate compound that can be used to treat COVID-19 as the antiviral activity of this molecule against SARS-CoV-2 has been reported. Rutin is a promising inhibitor of M^pro^ and other protein targets of the SARS-CoV-2 virus [30,31]. Rutin, a medicinally significant flavonoid, is one of nature’s finest antioxidants [32]. Notably, it has antiprotozoal [33], antibacterial [34], and antiviral properties [35]. Moreover, rutin binds effectively with the essential proteins of SARS-CoV-2. In one study, researchers stated that rutin has a strong binding pattern to the pocket of SARS-CoV-2 RNA-dependent RNA polymerase (RdRp), which may result in the strong inhibition of SARS-CoV-2 RdRp [32,36]. Furthermore, fumariline is an isoquinoline alkaloid [37] that has a good interaction with the targets M^pro^ and RBD. Additionally, two alkaloids (thalimonine and sophaline D) have demonstrated potential inhibitory activity against M^pro^ [38]. Drug-like alkaloids have been reported to have the potential to prevent SARS-CoV-2 cell entry through inhibition of spike glycoproteins [39].

Samarcandin, a natural sesquiterpene coumarin, is another molecule with high antiviral activity against RBD (−7.4 kcal/mol). Spectroscopic data show that samarcandin has strong similarity to badrakemin [40]. In the present study, badrakemin acetate was also a good inhibitor against RBD (−8.0 kcal/mol). Somniferine bound to RBD with a binding free energy of −6.7 kcal/mol. This compound was reported to have a high affinity for Mpro from SARS-CoV-2 in silico [41]. Somniferine is an alkaloid found in *Withania somnifera*, a multipurpose medicinal plant belonging to the family Solanaceae [42]. Additionally, withanolides are good candidates for COVID-19 treatment. Withanolides are a class of polyoxygenated steroid lactones that can be found in a wide range of plants [43]. In one study, withanolide Q was predicted to modulate the highest number of proteins, showed positive human intestinal absorption, and had the highest drug-likeness score. Similarly, withanolide D and withanolide G were predicted to have better binding affinity with SARS-CoV-2 papain-like cysteine protease (PL^pro^), withanolide M with M^pro^, and withanolide M with spike protein based on binding energy and the number of hydrogen bond interactions [44]. In the present study, withanone (−7.7 (kcal/mol) against RBD), withanolide A (−8.6 (kcal/mol) against M^pro^), and withanolide G (−7.4 (kcal/mol) against RBD) showed the best binding affinity to receptors among the withanolides (Table 1). Astragalin, a bioactive natural flavonoid, is known to possess antiviral activity [45]. In our study, astragalin showed the best binding affinity to M^pro^ (−8.8 kcal/mol) (Table 2).

The interaction types for both of the studied receptors are shown in Table 2 and Table 3. These compounds appeared to bind to the substrate binding site of Mpro, specifically His41–Cys145 catalytic dyad, except for chelidimerine and adlumidine. SARS-CoV-2 Mpro is a cysteine protease (CP) with a catalytic dyad in the active site Cys145/His41, similar to other CPs [46,47]. On the other hand, several researchers have previously reported that amino acid residues on RBD of SARS-CoV-2 that bind to hACE2 are Lys417, Tyr449, Gln493, Gly496, Gln498, Thr500, and Gly502 [48,49,50,51]. None of the compounds studied, however, interacted with these amino acids. On the other hand, several of these compounds interacted with the CR3022 epitope on the RBD with Ala372 and Phe374 [52]. The presence of these interactions on the CR3022 epitope allosterically perturbs ACE2 binding to the RBD [53].

In order from strongest to weakest, the intermolecular forces are as follows: ion–dipole, hydrogen bonding, dipole–dipole, and van der Waals forces. An abundance of hydrogen bonding occurred between our selected molecules and the two studied receptors (Table 2 and Table 3). Any molecule, be it a protein or ligand, is composed of atoms, while atoms are composed of a nucleus (with protons and neutrons) and electrons [54]. In protein–ligand docking, the conformations of ligands binding to receptor proteins were assessed, and the binding energies between protein–ligand pairs were quantified. Whenever a ligand interacts with a protein, electrons are involved in the formation of covalent or noncovalent bonds [55,56,57]. These pi-alkyl and pi-sulfur interactions belong to the broad category of noncovalent interactions [58]. In pi-alkyl interactions, a pi-electron cloud interacts with an aromatic group and the electron group of an alkyl group. In pi-sulfur interaction, the pi-electron cloud of an aromatic ring interacts with the lone pair of the electron cloud of the sulfur atom [59]. Pi-sigma interactions (pi-alkyl and pi-sulfur) are largely involved in charge transfer and help to intercalate drugs in the binding sites of receptors [60]. The result of this complex stability can be linked to pi-sigma interactions [61]. The presence of pi-sulfur and pi-alkyl interactions in the complex has been identified as a direction strain in the backbone of the drug responsible for normalizing the dipole moment of a drug through charge transfer with its neighboring amino acids [62].

In molecular docking models, hydrogen bond interactions, pi-pi stacking, and cation-pi stacking interactions are widely noticed [63]. The ligands with hydrophobic interactions significantly contribute to binding affinity. The lower frequency of hydrogen bonds reflects the fact that the binding pocket is more hydrophobic [64,65]. The protein–ligand binding free energy can be fully described by contributions from electrostatic, preorganized electrostatic, and nonelectrostatic interactions. The electrostatic contribution (including hydrogen bonding interactions) can be quantified with Coulomb’s potential [66,67], while the nonelectrostatic contribution can be quantified with Lennard-Jones’ potential. Preorganized electrostatic contribution can be quantified with Coulomb’s potential using geometries obtained by sampling the conformational space (molecular dynamics simulation) with the partial atomic charges of the ligand set to zero. The entropic contribution is implicitly included in the ensemble of geometries. The relative importance of electrostatic and nonelectrostatic contributions depends on the charge and shape complementarity of the interacting entities. Notably, preorganized electrostatic contribution considers the relative stability of properly oriented ligand binding groups in the protein and the bulk solvent. A conformation with more favorable electrostatic interactions has less favorable nonelectrostatic interactions, and vice versa [68,69,70].

The binding mechanisms involve cavity exploration, in which hydrophobic interactions contribute heavily to the binding affinity of the docked complex in a given solvent system AS they are entropy-driven interactions. Hydrophobic contacts include p-cation, p-p, and other nonspecific interactions [71]. These contacts are important for the folding of proteins to keep them stable and biologically active and reduce undesirable interactions with water [5]. Pi-pi T-shaped interaction involves an interaction of the pi-electron cloud between two aromatic groups in a T-shaped manner (i.e., the sidewise electron cloud of one ring and head-on electron cloud of other rings) [72]. This interaction occurs between chelidimerine, catechin gallate, pelargonidin-3 glucoside, astragalin, norsanguinarine, cyanidin 3,5-di-O-glucoside, cyanidin 3-O-rutinoside, and harpagoside with the M^pro^ receptor. This interaction occurs between these compounds and HisA:41 at the receptor. H-bonds, on the other hand, have a significant impact on the interaction. When more hydrogen bonds are formed with the amino acid residue, the stronger bonds will cause the energy score to be lower, while the bonds become more stable [73]. Hydrogen bonds are interactions between hydrogen atoms (H), which are covalently bonded with atoms such as fluorine (F), nitrogen (N), and oxygen (O) [74,75].

The predicted inhibition constant was evaluated for the selected compounds. The higher the IC50 value, the higher the inhibitory dose required to achieve the desired effect. As a result, it will further increase the drug candidate’s off-target probability, making it potentially toxic. Additionally, in this study, the higher the IC value, the lower was the binding free energy value. Alternatively, a high IC value indicates that a compound has a low affinity for its target. The compounds chosen for their high affinity for the studied receptors have an average toxicity of class IV or V (harmful or possibly harmful if swallowed), with the exception of withanone, which is classified as class II (fatal if swallowed). Numerous strategies can be implemented in drug design during the decision-making process to reduce the toxicity and metabolic instability of drug candidates.

A molecular dynamics simulation (MDS) study is frequently used to predict the stability of proteins and ligands [67]. The interaction between the best protein–ligand interaction obtained from AutoDock Vina was further examined in the MD study. The ligand-free complex (Apo) was used as a control system to compare the changes in protein stability after ligand binding. The RMSD of the backbone atoms from M^pro^ protein systems was analyzed to understand the structural deviations of the complexes. As shown in Figure 3A, while the chelidimerine and protein complexes had a stable RMSD profile at the initial time period (0–5 ns), they extended their RMSD profiles by exhibiting a high level of deviation from 5–10 ns and decreased their RMSD profile once again. This complex began to stabilize after 20 ns and maintained stability until 50 ns. The ligand-free Apo protein structure also had a stable profile but did not show excessive fluctuations as per chelidimerine at the initial phase. This might have occurred due to unstable conformational changes of the main protease at the initial phases following ligand binding.

Moreover, the RMSF values of the complexes were analyzed to understand the flexible amino acid residues across the proteins [76]. Figure 3B shows that the residues exhibited RMSF values lower than 1.2 nm over the entire simulation process, except for Glu47, Asp48, Met49, Leu50, Asn51, Pro52, Thr304, Phe305, and Gln 306 residues. These amino acids are not found in the pocket of M^pro^, which contains the amino acids His41, Cys145, His163, His164, and Glu166 in its active site [77]. Therefore, the Rg values of the simulated complexes were analyzed to examine the labile nature of the protein systems, where higher Rg values corelate with a more mobile nature of a protein and a lower Rg value indicates the stable nature of a system [78]. Figure 3C indicates that both Apo and ligand complexes had similar profiles from 0 to 30 ns. However, after that point, the Apo complexes increased their Rg values, indicating a more flexible nature. On the other hand, the Rg values of ligand complexes decreased, which highlighted the contracted nature of these complexes.

SASA is correlated with changes in protein surface area [79], where high SASA indicates the expansion of the surface area and lower SASA values indicate a decrease in protein volumes [80]. Figure 3D indicates that the SASA profiles of both systems were similar from the initial to final phases of the simulation, and no significant deviations in SASA were observed for these complexes.

The hydrogen bonds between proteins and ligands were also analyzed as they serve a vital role in defining the stable state of complexes [81]. As shown in Figure 3E, the hydrogen bond patterning of chelidimerine complexes was stable and did not significantly change in the simulation environments. Moreover, the strengths of interaction between the compounds and protein under study were computed in the form of nonbonded interaction energy. The average short-range Leonard-Jones interaction energies were calculated as −154,754 kJ/mol. The interaction energies seemed to be in the stable state and did not deviate. The low Lennard-Jones interaction energies were corelated with the improved stability and higher potency level of the systems.

A trajectory analysis of the RBD–somniferine complex was performed, and the results are presented in Figure 4. The RMSD values of the backbone atoms from the spike proteins were also analyzed, and Apo protein RMSD values increased after 5 ns. The RMSD values of Apo systems were similar from 5 to 50 ns and did not change significantly. Therefore, the ligand complex with somniferine had a higher degree of deviation after 5–20 ns but did not excessively fluctuate (as per Apo complexes at the initial phases). Somniferine then stabilized and maintained its integrity. The RMSF values of both systems were also analyzed. Lower fluctuations were found for nearly every amino acid residue in the spike protein, except Glu327, Thr328, Pro330, Asn331, Ile332, Lys528, and Lys529. The Rg profile of Apo protein was low at the initial phases (10–20 ns), and higher deviations were observed in these segments, which might be responsible for the higher level of mobility of these protein systems. Therefore, the Apo protein subsequently stabilized after 25 ns and maintained a stable profile for the remainder of the simulation. The RBD–somniferine complex also had initial fluctuations similar to that of Apo protein, which highlighted the labile nature from 0 to 20 ns; however, it reached a steady state thereafter.

The SASA values of both systems were stable at the initial phase from 0–20 ns. After 20–23 ns, the SASA profiles fluctuated but reached a steady state, which indicated no changes in protein surface area over the entirety of the simulations. The hydrogen bond pattern (between 1 and 2) and short-range average interaction energy (−125,068 kJ/mol) from the simulation trajectories were stable and did not fluctuate excessively.

## 4. Materials and Methods

### 4.1. Dataset

A total of 169 compounds from a variety of aromatic and medicinal plants were selected for this study. Table S1 shows the origin of each studied compound. These molecules were considered for molecular docking study.

### 4.2. Molecular Docking Analysis

#### 4.2.1. Ligand Preparation

In the present study, the structures of the compounds used as ligands were downloaded in structured data format (SDF) from PubChem (https://pubchem.ncbi.nlm.nih.gov/) and converted into pdb (protein data bank) format using Open Babel for further analysis [82]. The ligands were prepared by minimizing the energy and addition of hydrogen atoms and charges as well as setting the number of active torsions using AutoDock Tools [83]. The results of optimization were saved in pdbqt format.

#### 4.2.2. Receptor Preparation

The crystal structures of SARS-CoV-2 main protease (M^pro^) (PDB ID: 6LU7) and the 6YLA spike receptor-binding domain (RBD) (PDB ID: 6YLA) were retrieved from the RCSB Protein Data Bank (https://www.rcsb.org/) in pdb format. The macromolecules were separated from water molecules, heteroatoms, and nonstandard ligands in BIOVIA Discovery Studio v. 2020 and saved in pdbqt format.

#### 4.2.3. Determination of Active Sites

The active sites of the receptors were predicted using the Computed Atlas of Surface Topography of proteins (CASTp), available from http://sts.bioe.uic.edu/castp/index.html?2was [84].

#### 4.2.4. Molecular Docking and Visualization of Ligand–Receptor Interactions

The molecular docking was executed using AutoDock Vina and AutoDock Tools. For the docking with AutoDock Vina, the data (in pdbqt format) for the ligands and receptors were copied into the Vina folder. The configuration of Vina was typed in Notepad, saved as a txt file, and run via Windows command prompt. The calculation of docking results was viewed in Notepad. The best pose of the ligand conformation on the receptor was marked by the lowest binding free energy. The values were displayed in a log file in txt format. In the docking process using AutoDock Tools, the saved receptor (receptor. pdbqt) and ligand (ligan.pdbqt) files were opened in the Grid menu. The GridBox was set according to the specified binding site area, then the file was saved in gpf format. In the docking menu, the receptor and ligand files were opened, the Rigid Filename was set for docking, and then the docking parameters file was saved in dpf format. The files in gpf and dpf formats were moved in one folder. Autogrid and AutoDock commands were performed using the command prompt. Visualization of the positions and orientations of the ligands on the active sites of receptors, as well as the interactions of the amino acids, were performed in BIOVIA Discovery Studio v. 2020. These interactions were displayed in 2D and 3D conformations [85].

### 4.3. ADMET Analysis

Assessment of pharmacokinetic properties and druglike nature was carried out by predicting ADME parameters on SwissADME (http://www.swissadme.ch/) [86] and pkCSM (http://biosig.unimelb.edu.au/pkcsm/) [87]. Toxicity prediction of the studied compounds was carried out on ProTox-II (https://tox-new.charite.de/protox_II/) [88]. This web-based tool requires canonical SMILES as input, so the SMILES was retrieved via the PubChem database.

### 4.4. Calculation of Predicted IC_50_

AutoDock Tools 4.2 [83] was used to determine the IC50. As a result, the GridBox needed to be determined to frame the interaction area between the protein and the ligand. Analysis was divided into two activities: molecular initialization and grid running. The IC_50_ of each docked complex was contained in the control.dlg folder.

### 4.5. Molecular Dynamics Simulation

Gromacs v. 2020.4 software [89,90,91] was used to conduct MD simulations after achieving the desired conformation after docking. The protein (M^pro^ and RBD) and ligand topologies were created with the pdb2gmx module in Gromacs using the Charmm36-Jul2020 force field [92,93]. TIP3P [94,95] was used to model the water molecules, and ions were added thereafter. The ligand topologies were generated by the Charmm General Force Field (CGenFF). A dodecahedron box was used, with the protein complexes positioned at least 1.0 nm from the box edge. Sodium ions were added to neutralize the charge systems. The energy minimization of the simulation systems was conducted by 50,000 steps of the steepest descent minimization algorithm. The solvent and ion systems were equilibrated in two restrained phases. The reference temperature was 300 K for the 0.1 ns NVT ensemble, and the reference pressure was 1.0 bar for the 1 ns NPT ensemble. The time step of the simulation system was set as 2.0 fs, and an unrestrained MD simulation of the equilibrated systems was performed. The short-range van der Waals cut-off was 1.2 nm. A Berendsen thermostat was used for temperature coupling, and a Parrinello–Rahman barostat was used for pressure coupling [96,97]. Finally, an MD simulation was conducted for 50 ns. The calculation was performed for root-mean-square deviation (RMSD), root-mean-square fluctuation (RMSF), radius of gyration (Rg), solvent accessible surface area (SASA), and intermolecular hydrogen bond analyses from the trajectory. Short-range protein–ligand interaction energy was calculated using the Lennard-Jones potential.

## 5. Conclusions

Currently, the search for new molecules with a preservative power of natural origin is based on ethnobotanical studies, which make it possible to conduct inventories of plants. With regard to phytochemical and pharmacological studies as well as other scientific endeavors, the importance of using medicinal plants has pushed researchers to seek molecules that can prevent SARS-CoV-2 infection. The results of molecular docking are highly satisfactory, and we discovered 20 molecules that are very interesting from both chemical and biological perspectives. Therefore, we propose these molecules as inhibitors of the SARS-CoV-2 M^pro^ and RBD receptors. The synthesis of these molecules and evaluation of their in vitro and in vivo activity against SARS-CoV-2 is worthy of further clinical study.

## Figures and Tables

**Table 1:** Binding analysis of the ligands against SARS-CoV-2 receptors using AutoDock Tools.

Ligands’ Chemical Name	PubChem ID	Binding Affinity to the Receptors (kcal/mol)	
		6LU7	6YLA
Lopinavir	92727	−5.2	-
Chelidimerine	190990	−10.2	−8.2
Withanolide G	21679023	−8.6	−8.4
Badrakemin acetate	1771505	−8.6	−8.0
Samarcandin	71587098	−8.5	−7.4
Catechin gallate	6419835	−8.6	−6.1
Somniferine	14106343	−8.3	−6.7
Withanone	21679027	−8.2	−7.8
Adlumidine	120734	−8.2	−6.8
Pelargonidin 3-glucoside	443648	−8.1	−6.2
Norsanguinarine	97679	−7.5	−7.0
Sanguinarine	5154	−7.7	−6.8
Fumariline	159888	−7.8	−6.4
Astragalin	5282102	−7.9	−4.4
Rutin	5280805	−7.4	−4.1
Cyanidin 3,5-di-O-glucoside	441688	−6.9	−2.8
Cyanidin 3-O-rutinoside	441674	−6.9	−4.0
Kaempferitrin	5486199	−6.1	−4.6
Harpagoside	5281542	−6.1	−3.9
Pinoresinol 4-O-b-d-glucopyranoside	486614	−4.9	−7.1

**Table 2:** Molecular interactions between the best ligands and the receptor main protease (6LU7).

Ligand	Interacting Residues	Category	Type of Interaction
Lopinavir	Glu:A166	H-bond	Conventional
	Asn:A142	H-bond	Conventional
	Gln:A189	H-bond	Conventional
	Gly:A143	H-bond	Conventional
	Asn:A142	H-bond	Carbon
	Met:A49	Hydrophobic	Pi-sigma
	Pro:A168	Hydrophobic	Alkyl/pi-alkyl
	His:A163	Hydrophobic	Alkyl/pi-alkyl
	Cys:A145	Hydrophobic	Alkyl/pi-alkyl
	His:A163	Hydrophobic	Pi-sulfur
	Cys:A145	Hydrophobic	Pi-sulfur
Chelidimerine	Cys:A145	H-bond	Pi-donor/carbon
	Phe:A140	H-bond	Pi-donor/carbon
	His:A41 (2)	Hydrophobic	Pi-pi T-shaped
	Met:A49	Hydrophobic	Alkyl/pi-alkyl
	Cys:A145	Hydrophobic	Alkyl/pi-alkyl
	Met:A165 (3)	Hydrophobic	Alkyl/pi-alkyl
	Pro:A168 (2)	Hydrophobic	Alkyl/pi-alkyl
	Asn:A142	Hydrophobic	Pi-sigma
Catechin gallate	Glu:A166	H-bond	Conventional
	Asp:A187	H-bond	Conventional
	Gln:A192	H-bond	Conventional
	Cys:A145	H-bond	Conventional
	Leu:A141	H-bond	Conventional
	His:A163	H-bond	Conventional
	Phe:A140	H-bond	Conventional
	Met:A165 (3)	Hydrophobic	Alkyl/pi-alkyl
	Pro:A168	Hydrophobic	Alkyl/pi-alkyl
	His:A41	Hydrophobic	Pi-pi T-shaped
	Cys:A145	Hydrophobic	Pi-sulfur
Badrakemin acetate	His:A41	H-bond	Conventional
	Gln:A192	H-bond	Conventional
	Cys:A145 (3)	Hydrophobic	Alkyl/pi-alkyl
	His:A163 (2)	Hydrophobic	Alkyl/pi-alkyl
	His:A41	Hydrophobic	Alkyl/pi-alkyl
	Met:A165 (2)	Hydrophobic	Pi-sulfur
	His:A41	Hydrophobic	Pi-sigma
Withanolide G	Asn:A142	H-bond	Conventional
	Gly:A143	H-bond	Conventional
	Glu:A166	H-bond	Conventional
	Asn:A142	H-bond	Carbon
	Cys:A145 (3)	Hydrophobic	Alkyl/pi-alkyl
	His:A163	Hydrophobic	Alkyl/pi-alkyl
	Pro:A168 (2)	Hydrophobic	Alkyl/pi-alkyl
	Met:A165	Hydrophobic	Alkyl/pi-alkyl
Samarcandin	Cys:A145	H-bond	Conventional
	Gly:A143	H-bond	Conventional
	Glu:A166 (2)	H-bond	Conventional
	Cys:A145	H-bond	Pi-donor
	Leu:A167	Hydrophobic	Alkyl
	Pro:A168 (2)	Hydrophobic	Alkyl
Somniferine	Gln:A189	H-bond	Conventional
	Glu:A166 (2)	H-bond	Conventional
	Met:A165	H-bond	Carbon
	His:A41	Hydrophobic	Alkyl/pi-alkyl
	Cys:A145	Hydrophobic	Alkyl/pi-alkyl
	Pro:A168	Hydrophobic	Alkyl/pi-alkyl
Adlumidine	Glu:A166	H-bond	Conventional
	Cys:A145	H-bond	Conventional
	Glu:A166	H-bond	Carbon
	Leu:A167	H-bond	Carbon
	Phe:A140	H-bond	Carbon
	Glu:A166 (2)	Hydrophobic	Pi-anion
	Cys:A145)	Hydrophobic	Pi-alkyl
Withanone	Glu:A166	H-bond	Conventional
	Cys:A145	H-bond	Conventional
	Gly:A143	H-bond	Conventional
	Ser:A144	H-bond	Conventional
	His:A41 (2)	Hydrophobic	Alkyl/pi-alkyl
	Met:A49	Hydrophobic	Alkyl/pi-alkyl
	Cys:A145 (2)	Hydrophobic	Alkyl/pi-alkyl
Pelargonidin-3 glucoside	His:A163	H-bond	Conventional
	His:A164	H-bond	Conventional
	Glu:A166 (2)	H-bond	Conventional
	Thr:A190	H-bond	Conventional
	Gln:A189	Hydrophobic	Pi-sigma
	His:A41	Hydrophobic	Pi-pi T-shaped
	Pro:A168	Hydrophobic	Pi-alkyl
	Met:A165	Hydrophobic	Pi-alkyl
	Met:A165 (2)	Hydrophobic	Pi-sulfur
Astragalin	Gln:A189 (2)	H-bond	Conventional
	Tyr:A54	H-bond	Conventional
	Glu:A166 (2)	H-bond	Conventional
	Thr:A190	H-bond	Conventional
	Met:A165 (3)	Hydrophobic	Pi-alkyl
	Met:A49	Hydrophobic	Pi-alkyl
	His:A41	Hydrophobic	Pi-pi T-shaped
Fumariline	Glu:A166	H-bond	Conventional
	Glu:A166	H-bond	Carbon
	Gln:A192	H-bond	Carbon
	Leu:A167	H-bond	Carbon
	Gln:A189	Hydrophobic	Amide-pi stacked
	Pro:A168	Hydrophobic	Pi-sigma
	Leu:A167	Hydrophobic	Pi-alkyl
	Pro:A168	Hydrophobic	Pi-alkyl
	Met:A165	Hydrophobic	Pi-alkyl
Sanguinarine	Tyr:A54	H-bond	Conventional
	Met:A49	H-bond	Carbon
	Asp:A187	H-bond	Carbon
	Cys:A145	H-bond	Carbon
	Glu:A166	H-bond	Carbon
	Met:A49 (2)	Hydrophobic	Alkyl/pi-alkyl
	Cys:A145 (2)	Hydrophobic	Alkyl/pi-alkyl
	His:A163	Hydrophobic	Pi-cation
Norsanguinarine	His:A163	H-bond	Conventional
	Leu:A141	H-bond	Carbon
	Ser:A144	H-bond	Carbon
	Met:A49 (2)	Hydrophobic	Pi-alkyl
	Cys:A145	Hydrophobic	Pi-alkyl
	Met:A165	Hydrophobic	Pi-alkyl
	His:A41 (2)	Hydrophobic	Pi-pi T-shaped
	Met:A165	Hydrophobic	Pi-sigma
Rutin	Asp:A187	H-bond	Conventional
	His:A41	H-bond	Conventional
	Arg:A188	H-bond	Conventional
	Thr(A190)	H-bond	Conventional
	Glu:A166 (2)	H-bond	Conventional
	Glu:A166	H-bond	Carbon
	Pro:A168	H-bond	Carbon
	Cys:A145 (2)	Hydrophobic	Pi-cation/pi-sulfur
	Met:A49	Hydrophobic	Pi-cation/pi-sulfur
	His:A49	Hydrophobic	Pi-cation/pi-sulfur
	Met:A165	Hydrophobic	Pi-alkyl
Cyanidin 3,5-di-O-glucoside	Gln:A192	H-bond	Conventional
	Glu:A166 (2)	H-bond	Conventional
	Gly:A143	H-bond	Conventional
	Leu:A141	H-bond	Conventional
	Glu:A166	H-bond	Carbon
	His:A41	Hydrophobic	Pi-pi T-shaped
	Gln:A189	Hydrophobic	Pi-sigma
	Met:A165 (2)	Hydrophobic	Pi-alkyl
	Pro:A168	Hydrophobic	Pi-alkyl
	Met:A165 (2)	Hydrophobic	Pi-sulfur
	His:A164	Unfavorable	Donor-donor
Cyanidin 3-O-rutinoside	Asn:A142 (2)	H-bond	Conventional
	Cys:A145	H-bond	Conventional
	His:A164	H-bond	Conventional
	Asp:A187	H-bond	Conventional
	Met:A49	H-bond	Conventional
	Thr:A190	H-bond	Conventional
	Gln:A189	H-bond	Carbon
	Met:A165 (2)	Hydrophobic	Pi-alkyl
	His:A41	Hydrophobic	Pi-pi T-shaped
	His:A163	Unfavorable	Donor-donor
	Gln:A192	Unfavorable	Donor-donor
Kaempferitrin	Ser:A144	H-bond	Conventional
	Cys:A145	H-bond	Conventional
	Leu:A141	H-bond	Conventional
	Glu:A166	H-bond	Conventional
	Asn:A142	H-bond	Conventional
	Glu:A166	H-bond	Pi-donor
	Met:A49	Hydrophobic	Alkyl/pi-alkyl
	Ala:A191	Hydrophobic	Alkyl/pi-alkyl
	Met:A165	Hydrophobic	Pi-sulfur
	Glu:A166	Hydrophobic	Pi-lone pair
Harpagoside	Glu:A166 (3)	H-bond	Conventional
	Asn:A142	H-bond	Conventional
	Leu:A141	H-bond	Carbon
	Met:A49	Hydrophobic	Pi-sulfur
	Met:A165	Hydrophobic	Pi-sulfur
	His:A41	Hydrophobic	Pi-pi T-shaped

**Table 3:** Molecular interactions between the best ligands and the receptor-binding domain (6YLA).

Ligand	Interacting Residues	Category	Type of Interaction
Withanolide G	Asn:E370	H-bond	Conventional
	Asp:C66	H-bond	Conventional
	Arg:C67	H-bond	Conventional
	Gln:C85	H-bond	Conventional
	Val:E367 (4)	Hydrophobic	Alkyl/pi-alkyl
	Phe:E374	Hydrophobic	Alkyl/pi-alkyl
Chelidimerine	Ser:E371	H-bond	Carbon
	Asp:E364	H-bond	Carbon
	Val:E367 (4)	Hydrophobic	Alkyl/pi-alkyl
	Phe:E374	Hydrophobic	Pi-sigma
	Phe:E338	Hydrophobic	Pi-sigma
Badrakemin acetate	Glu:E340	H-bond	Conventional
	Gly:E339	H-bond	Conventional
	Asn:E343	H-bond	Pi-donor
	Val:E367 (3)	Hydrophobic	Alkyl/pi-alkyl
	Leu:E335 (3)	Hydrophobic	Alkyl/pi-alkyl
	Leu:E368	Hydrophobic	Alkyl/pi-alkyl
	Phe:E338 (2)	Hydrophobic	Alkyl/pi-alkyl
	Asn:E343	Hydrophobic	Pi-lone pair
Withanone	Gly:E339	H-bond	Pi-donor
	Trp:E436 (3)	Hydrophobic	Alkyl/pi-alkyl
	Leu:E368 (2)	Hydrophobic	Alkyl/pi-alkyl
	Phe:E342 (2)	Hydrophobic	Alkyl/pi-alkyl
	Phe:E374	Hydrophobic	Alkyl/pi-alkyl
	Val:E367	Hydrophobic	Alkyl/pi-alkyl
	Ser:E371	Unfavorable	Donor-donor
Samarcandin	Asn:E343	H-bond	Conventional
	Asp:E364	H-bond	Conventional
	Leu:E335	Hydrophobic	Pi-alkyl
	Val:E367	Hydrophobic	Pi-alkyl
	Val:E367	Hydrophobic	Pi-sigma
	Phe:E338	Hydrophobic	Pi-sigma
Pinoresinol 4-O-b-d-glucopyranoside	Asp:E364	H-bond	Conventional
	Cys:E336	H-bond	Conventional
	Asn:E343 (2)	H-bond	Conventional
	Ser:E371	H-bond	Conventional
	Val:E367 (2)	Hydrophobic	Pi-alkyl
	Leu:E368	Hydrophobic	Pi-alkyl
	Val:E367	Hydrophobic	Pi-sigma
	Phe:E338	Hydrophobic	Pi-sigma
Norsanguinarine	Asp:C66	H-bond	Conventional
	Val:E367	H-bond	Conventional
	Asp:C66	H-bond	Carbon
	Arg:C67	Hydrophobic	Pi-alkyl
	Val:E367	Hydrophobic	Pi-alkyl
	2Asn:E370	Hydrophobic	Amide-pi stacked
Sanguinarine	Asn:E343	H-bond	Conventional
	Leu:E335	Hydrophobic	Alkyl/pi-alkyl
	Val:E367 (2)	Hydrophobic	Alkyl/pi-alkyl
	Val:E367	Hydrophobic	Pi-sigma
	Phe:E342	Hydrophobic	Pi-pi stacked
Adlumidine	Asn:E343	H-bond	Conventional
	Val:E367	H-bond	Carbon
	Leu:E368	H-bond	Carbon
	Val:E367	Hydrophobic	Pi-sigma
	Phe:E374	Hydrophobic	Pi-alkyl
	Phe:E374	Hydrophobic	Pi-pi T-shaped
	Trp:E436	Hydrophobic	Pi-pi T-shaped
	Phe:E338	Hydrophobic	Pi-pi T-shaped
Somniferine	Ser:E371 (2)	H-bond	Conventional
	Asn:E343	H-bond	Carbon
	Trp:E436 (3)	Hydrophobic	Alkyl/pi-alkyl
	Val:E367	Hydrophobic	Alkyl/pi-alkyl
	TrpE436	Hydrophobic	Pi-pi stacked
	Asn:E343	Unfavorable	Donor-donor
Fumariline	Gly:E339	H-bond	Conventional
	Leu:E335 (2)	Hydrophobic	Alkyl/pi-alkyl
	Val:E367 (2)	Hydrophobic	Alkyl/pi-alkyl
	Phe:E338	Hydrophobic	Pi-sigma
Pelargonidin-3 glucoside	Asn:E343 (2)	H-bond	Conventional
	Ser:E371	H-bond	Conventional
	Asn:E364:	H-bond	Conventional
	Cys:E336	H-bond	Conventional
	Phe:E338	Hydrophobic	Pi-sigma
	Val:E367	Hydrophobic	Pi-sigma
	Val:E367 (2)	Hydrophobic	Pi-alkyl
	Leu:E368	Hydrophobic	Pi-alkyl
Catechin gallate	Ser:E375	H-bond	Conventional
	Asn:E440 (2)	H-bond	Conventional
	Asn:E437	H-bond	Conventional
	Arg:E509	H-bond	Conventional
	Phe:E342	H-bond	Conventional
	Trp:E436	Hydrophobic	Pi-alkyl
	Leu:E441	Hydrophobic	Pi-sigma
	Trp:E436	Hydrophobic	Pi-pi T-shaped

**Table 4:** Pharmacological properties of the top potential candidates derived from swissADME, ProTox-II, and pkCSM.

Parameters	Chelidimerine	Withanolide G	Badrakemin Acetate	Samarcandin	Catechin Gallate	Somniferine	Withanone	Cyanidin 3-O-Rutinoside
Molecular weight	720.7 g/mol	454.6 g/mol	424.5 g/mol	400.5 g/mol	442.4 g/mol	608.7 g/mol	470.6 g/mol	595.5 g/mol
H-bond acceptor	11	5	5	5	10	9	6	14
H-bond donor	0	2	0	2	7	2	2	10
CNS	−2.718	−2.894	−1.638	−2.044	−3.743	−3.073	−2.719	−4.943
CYP2D6 substrate	No	No	No	No	No	No	No	No
CYP3A4 substrate	Yes	Yes	Yes	Yes	No	Yes	Yes	No
CYP1A2 inhibitor	No	No	No	No	No	No	No	No
CYP2C19 inhibitor	No	No	Yes	No	No	No	No	No
CYP2C9 inhibitor	No	No	Yes	No	No	No	No	No
CYP2D6 inhibitor	No	No	No	No	No	No	No	No
CYP3A4 inhibitor	No	No	Yes	No	No	No	No	No
Carcinogenicity	Carcinogenic	Carcinogenic	Noncarcinogenic	Noncarcinogenic	Noncarcinogenic	Noncarcinogenic	Noncarcinogenic	Noncarcinogenic
Hepatotoxicity	No	No	No	Yes	No	No	No	No
p-glycoprotein substrate	Yes	Yes	No	Yes	No	Yes	Yes	No
Acute oral toxicity	Class IV, LD50 1408 mg/kg	Class IV, LD50 400 mg/kg	Class V, LD50 3200 mg/kg	Class V, LD50 3200 mg/kg	Class IV, LD50 1000 mg/kg	Class IV, LD50 1100 mg/kg	Class II, LD50 7 mg/kg	Class V, LD50 5000 mg/kg
Lipinski rule of five	No	Yes	Yes	Yes	Yes	Yes	Yes	No

**Table 5:** Predicted half-maximal inhibitory concentration (IC50) values.

Ligands’ Chemical Name	Predicted IC_50_	
	6LU7	6YLA
Lopinavir	136.22 µM	-
Chelidimerine	25.90 nM	843.38 nM
Withanolide G	455.10 nM	614.03 nM
Badrakemin acetate	478. 95 nM	1.23 µM
Samarcandin	578.21 nM	3.57 µM
Catechin gallate	433.52 nM	29.17 µM
Somniferine	776.89 nM	12.30 µM
Withanone	895.84 nM	1.68 µM
Adlumidine	953.86 nM	9.48 µM
Pelargonidin 3-glucoside	1.06 µM	25.10 µM
Norsanguinarine	2.91 µM	6.60 µM
Sanguinarine	2.05 µM	10.24 µM
Fumariline	1.77 µM	19.31 µM
Astragalin	1.49 µM	508.13 µM
Rutin	3.59 µM	894.70 µM
Cyanidin 3,5-di-O-glucoside	8.43 µM	1.09 mM
Cyanidin 3-O-rutinoside	8.12 µM	8.59 mM
Kaempferitrin	31.75 µM	381.16 mM
Harpagoside	31.75 µM	1.30 mM
Pinoresinol 4-O-b-d-glucopyranoside	245.63 µM	5.53 µM

## Data Availability

Data regarding this article will be provided upon request.

## Supplementary Materials

The following are available online. Table S1: Plants chemical composition with their PubChem ID, Table S2: Binding analysis of the ligands against SARS-CoV-2 receptors using AutoDock Vina.
