# Supplementary material for: In Silico Evaluation of Iranian Medicinal Plant Phytoconstituents as Inhibitors against Main Protease and the Receptor-Binding Domain of SARS-CoV-2
Source: Molecules. 2021 Sep 21;26(18):5724. doi: 10.3390/molecules26185724 (PMC8470205; doi:10.3390/molecules26185724)
Supplement: Supplementary file 1 [file molecules-26-05724-s001.zip › molecules-1295447-supplementary.pdf]

Supplementary Materials

Article

In Silico Evaluation of Iranian Medicinal Plant Phytoconstituents as Inhibitors against Main Protease and the Receptor-Binding Domain of SARS-CoV-2

Seyyed Sasan Mousavi[1], Akbar Karami[1], Tahereh Movahhed Haghighi[1], Sefren Geiner Tumilaar[2], Fatimawali[2,3], Rinaldi Idroes[4], Shafi Mahmud[5], Ismail Celik[6], Duygu Ag ˇagündüz[7], Trina EkawatiTallei[3,8,*], Talha Bin Emran[9,*] and Raffaele Capasso[10,*]

[1]Department of Horticultural Science, Faculty of Agriculture, Shiraz University, Shiraz71454, Iran; seyedsasanmousavi66@gmail.com (S.S.M.); akbarkarami@shirazu.ac.ir (A.K.); tmovahhed@gmail.com (T.M.H.)
[2]Pharmacy Study Program, Faculty of Mathematics and Natural Sciences, Sam Ratulangi University, Manado, North Sulawesi 95115, Indonesia; sefrentumilaar1@gmail.com (S.G.T.); fatimawali@unsrat.ac.id (F.)
[3]The University Center of Excellence for Biotechnology and Conservation of Wallacea, Sam Ratulangi University, Manado, North Sulawesi 95115, Indonesia
[4]Department of Pharmacy, Faculty of Mathematics and Natural Sciences, Universitas Syiah Kuala, Kopelma Darussalam, Banda Aceh 23111, Indonesia; rinaldi.idroes@unsyiah.ac.id (R.I.)
[5]Microbiology Laboratory, Department of Genetic Engineering and Biotechnology, University of Rajshahi, Rajshahi 6205, Bangladesh; shafimahmudfz@gmail.com (S.M.)
[6]Department of Pharmaceutical Chemistry, Faculty of Pharmacy, Erciyes University, Kayseri 38039, Turkey; ismailcelik@erciyes.edu.tr (I.C.)
[7]Department of Nutrition and Dietetics, Faculty of Health Sciences, Gazi University, Emek, Ankara 06490, Turkey; duyguturkozu@gazi.edu.tr (D.A.)
[8]Department of Biology, Faculty of Mathematics and Natural Sciences, Sam Ratulangi University, Manado, North Sulawesi 95115, Indonesia
[9]Department of Pharmacy, BGC Trust University Bangladesh, Chittagong 4381, Bangladesh
[10]Department of Agricultural Sciences, University of Naples Federico II, 80055 Portici, (Naples) Italy

* Correspondence: rafcapas@unina.it (R.C); trina_tallei@unsrat.ac.id (T.E.T.); talhabmb@bgctub.ac.bd (T.B.E.) Tel.: +39.081678664 (R.C.); +662-811-4314-880 (T.E.T.); +88-01819-942214 (T.B.E.)

Table S1. Plants chemical composition with their PubChem ID.

No.	Plants Name	Compounds
1	*Allium sativum*	Ajoene<br>Allicin<br>Alliin<br>Methyl allyl thiosulfinate<br>Allitridin<br>Garlicin
2	*Artemisia annua*	Borneol<br>Stigmasterol<br>p-Hydroxyacetophenone
3	*Artemisia capillaris*	Camphor<br>Scoparone<br>Stigmasterol
4	*Artemisia princeps var. orientalis*	α-thujone<br>β-sitosterol<br>Stigmasterol<br>Chlorogenic acid
5<br>6<br>7	*Berberis holstii*<br>*Berberis vulgaris*<br>*Berberis lycium*	Berberine
8	*Capparis sinaica*	Rutin<br>Quercetin<br>Isoquercetin
9	*Capparis spinosa*	Rutin<br>Quercetin<br>Kaempferol
10	*Carum copticum*	O-cymene<br>Terpinolene<br>Thymol
11	*Ephedra sinica*	Lupeol<br>-2Nonaprenyl-6-methoxyphenol<br>Glycodeoxycholic acid<br>Ledene<br>L-methylephedrin<br>L-ephedrine<br>D-pseudoephedrine
12	*Ferula sp.*	Badrakemin acetate<br>Kellerin<br>Samarcandin
13	*Fumaria indica*	Reticuline<br>Norjuziphine<br>Sanguinarine<br>Norsanguinarine<br>Chelidimerine<br>Fumarophycine<br>Corpaine<br>Sibiricine<br>Fumariline

		Berberine<br>Parfumine<br>Dihydrofumariline<br>α-Hydrastine<br>Bicuculline<br>Adlumidine<br>Bulbocapnine<br>Sinactine<br>Palmatine<br>Dehydrocavidine<br>Cularicine<br>Oxocularine
14	*Glycyrrhiza glabra*	Glycyrrhizate<br>Glycyrrhizin<br>Glycyrrhetinic acid<br>Glabridin<br>Glycycoumarin<br>Glycyrol<br>Liquiritigenin<br>Isoliquiritigenin<br>Licochalcone A<br>Ergosterol<br>Licochalcone B<br>Stigmasterol
15	*Heracleum pastinaca*	Rutin<br>Quercetin<br>α-pinene<br>Limonene<br>Isorhamnetin<br>Astragalin
16	*Heracleum rigens*	Rutin<br>Quercetin<br>α-pinene<br>Limonene<br>bornyl acetate
17	*Hibiscus sabdariffa*	Rutin<br>Quercetin<br>Cyanidin<br>protocatechuic acid<br>Gallocatechin gallate
18	*Hibiscus schizopetalus*	Cyanidin 3-O-rutinoside<br>Rutin<br>Quercetin<br>Gallocatechin gallate<br>protocatechuic acid<br>Cyanidin-3,5 di-O-glucoside<br>Gossypetin<br>Gossypetin 3 glucuronide<br>Delphinidin

		Hibicuslide C<br>Hibiscetin<br>Hibiscetin glucoside<br>Hibiscus acid<br>Hibiscus lactone<br>Malvidin<br>Petunidin<br>Pelargonidin<br>Pelargonidin-3 glucoside<br>Kaempferitrin<br>Myricetin<br>Apigenin<br>Catechin gallate<br>Cleomiscosin A<br>Stearic acid<br>Linoleic acid<br>Palmitic acid<br>Ferulic acid<br>β-propiolactone
19	*Myrtus commuins*	Sabinene
20	*Nepeta cataria*	Limonene<br>Pulegone<br>Menthone<br>Rosmarinic acid<br>Caryophyllene oxide<br>Carvone
21	*Nepeta tenuifolia*	Caryophyllene oxide<br>Limonene<br>Pulegone<br>Caryophyllene oxide<br>Rosmarinic acid<br>Menthone<br>Carveol<br>Isopulegone<br>β-Myrcene<br>Menthofuran<br>Piperitenone<br>Schizonepetin<br>Verbenone<br>γ-Terpinene
22	*Oliveria decumbens Vent.*	Myristicin
23	*Origanum acutidens*	α-pinene<br>Caryophyllene oxide<br>Caryophyllene oxide
24	*Polygonum minus*	Ellagic acid<br>Methyl gallate<br>Caffeic acid<br>Lobeline
25	*Pulicaria sp.*	p-cymene

26	*Rheum emodi*	Emodin
27	*Rheum palmatum*	Aloe-emodin
28	*Rheum tanguticum*	Rhein<br>Chrysophanol<br>Physcion<br>Sennoside A
29	*Salvia officinalis*	Protocatechuic acid<br>Rosmarinic acid
30	*Salvia plebeia*	Ursolic acid<br>Rosmarinic acid<br>Hispidulin<br>Daucosterol<br>Protocatechuic acid<br>Nepetin<br>Rosmarinic acid methyl ester<br>Methyl p-hydroxyphenyllactate<br>Luteolin<br>Citrusin
31	*Saxifraga spinulosa*	Pyrogallol<br>Gallocatechin gallate
32	*Smilax china*	Dihydrokaempferol
33	*Smilax glabra*	Resveratrol<br>Oxyresveratrol
34	*Teucrium polium*	Cepharanthine<br>Troxerutin<br>Limonene<br>Deoxyloganin tetraacetate<br>Rhoifolin<br>Sericetin diacetate<br>Carapin-8(9)-ene<br>Selinidin<br>Harpagoside<br>-8Epiiridodial glucoside tetraacetate<br>Larixol Acetate<br>Valtratum<br>Triptonide<br>Koparin<br>Dihydrosamidin<br>Khayanthone<br>-10Hydroxyloganin<br>β-caryophyllene<br>germacrene D<br>β-Thujone
35	*Urtica dioica*	N-acetylglucosamine-
36	*Withania somnifera*	Withanoside V<br>Somniferine<br>Withaferin A<br>Withanolide D<br>Withanolide G

		Withanolide M<br>-12deoxywithastramonolide<br>Withanolide A<br>Withanone<br>Withanolide B<br>Viscosalactone B<br>-27hydroxywithanone<br>dihydrowithaferin A<br>withanolide
37	*Zataria multiflora*	Thymol<br>Rosmarinic acid<br>Carvacrol
38	*Zhumeria majdae*	Rosmarinic acid

Table S2. Binding analysis of the ligands against SARS-CoV-2 receptors using AutoDock Vina.

No.	Ligands' Chemical Name	PubChem ID	Binding affinity to the receptors (kcal/mol)	
			6LU7	6YLA
1	Chelidimerine	190990	-10.3	-7.5
2	Rutin	5280805	-9	-6.4
3	Fumariline	159888	-9	-7.4
4	Catechin gallate	6419835	-9	-6.5
5	Adlumidine	120734	-8.9	-7.1
6	Pelargonidin-3 glucoside	443648	-8.9	-6.5
7	Astragalin	5282102	-8.8	-6.7
8	Somniferine	14106343	-8.8	-7.9
9	Cyanidin-3 O-rutinoside	441674	-8.7	-6.8
10	Cyanidin-3,5 di-O-glucoside	441688	-8.7	-6.8
11	Withanolide A	11294368	-8.6	-6.7
12	Bulbocapnine	12441	-8.5	-6.5
13	27-Hydroxywithanone	21574483	-8.5	-6.9
14	Gallocatechin gallate	5276890	-8.5	-7.3
15	Sanguinarine	5154	-8.4	-7.8
16	Dihydrofumariline	605542	-8.4	-6.7
17	Withanolide M	25090669	-8.4	-6.9
18	Isoquercetin	5280804	-8.3	-6.7
19	Sibiricine	632652	-8.3	-6.9
20	Gossypetin 3- glucuronide	44259994	-8.3	-6.2
21	12-Deoxywithastramonolide	44576309	-8.3	-6.7
22	Norsanguinarine	97679	-8.2	-7.7
23	Bicuculline	10237	-8	-6.7
24	Rosmarinic acid	5281792	-8	-6.6
25	Citrusin	101127372	-8	-6.9
26	Rhoifolin	5282150	-8	-6.9
27	Sericetin diacetate	4574925	-8	-5.8
28	Withaferin A	265237	-8	-6.4
29	Dihydrowithaferin A	15411208	-8	-6.4
30	Glycyrrhetinic acid	3230	-7.9	-7.1
31	Licochalcone A	5318998	-7.9	-6
32	Carapin-8-(9)-Ene	6708728	-7.9	-6.4
33	Withanolide B	14236711	-7.9	-6.9
34	Glycodeoxycholic acid	3035026	-7.8	-7.1
35	Badrakemin acetate	1771505	-7.8	-7.5
36	Glabridin	124052	-7.8	-7.2
37	Troxerutin	5486699	-7.8	-5.4
38	Withanolide D	161671	-7.8	-7.3
39	Viscosalactone B	57403080	-7.8	-6.8
40	Berberine	2353	-7.7	-6.4
41	Kaempferol	5280863	-7.7	-5.9
42	Sinactine	5321317	-7.7	-6.3

43	Cularicine	442201	-7.7	-6.5
44	Liquiritigenin	114829	-7.7	-6
45	Pelargonidin	440832	-7.7	-6
46	Kaempferitrin	5486199	-7.7	-7.4
47	Apigenin	5280443	-7.7	-6.5
48	Cleomiscosin A	442510	-7.7	-6.3
49	Ursolic acid	64945	-7.7	-6.6
50	Hispidulin	5281628	-7.7	-5.9
51	Withanolide G	21679023	-7.7	-7.4
52	Pinoresinol-4 O-b-D-glucopyranoside	486614	-7.6	-7.4
53	α-Hydrastine	442247	-7.6	-6.3
54	Harpagoside	5281542	-7.6	-7.4
55	Triptonide	65411	-7.6	-7.3
56	Koparin	5318834	-7.6	-6.2
57	Withanoside V	10700345	-7.6	-6.4
58	Withanolide	53477765	-7.6	-7.2
59	Quercetin	5280343	-7.5	-6.1
60	Kellerin	40580807	-7.5	-7.1
61	Fumarophycine	631930	-7.5	-5.8
62	Oxocularine	73044	-7.5	-6.1
63	Isoliquiritigenin	638278	-7.5	-6.3
64	Licochalcone B	5318999	-7.5	-6.6
65	Petunidin	441774	-7.5	-6.2
66	Ellagic acid	5281855	-7.5	-6.1
67	Rhein	10168	-7.5	-6.6
68	Nepetin	5317284	-7.5	-6.2
69	Withanone	21679027	-7.5	-7.7
70	Lupeol	259846	-7.4	-6.7
71	Glycyrol	5320083	-7.4	-6.4
72	Gossypetin	5280647	-7.4	-6.2
73	Hibiscetin	15559735	-7.4	-6.1
74	Myricetin	5281672	-7.4	-6
75	Lobeline	101616	-7.4	-7.1
76	Luteolin	5280445	-7.4	-6
77	Dihydrokaempferol	122850	-7.4	-6.1
78	Selinidin	668079	-7.4	-6.1
79	8-Epiiridodial glucoside tetraacetate	443339	-7.4	-5.4
80	Samarcandin	71587098	-7.3	-7.4
81	Corpaine	442197	-7.3	-6.6
82	Glycycoumarin	5317756	-7.3	-6.2
83	Ergosterol	444679	-7.3	-6.3
84	Isorhamnetin	5281654	-7.3	-6
85	Delphinidin	128853	-7.3	-6
86	Hibiscetin glucoside	44259992	-7.3	-5.9
87	Rosmarinic acid methyl ester	3012090	-7.3	-6.3
88	Cepharanthine	10206	-7.3	-7.2

89	Parfumine	185623	-7.2	-6.7
90	Palmatine	19009	-7.2	-6.2
91	Cyanidin	128861	-7.2	-6.2
92	Deoxyloganin tetraacetate	443329	-7.2	-5.1
93	Stigmasterol	5280794	-7.1	-6.8
94	Chlorogenic acid	1794427	-7.1	-6.4
95	Malvidin	159287	-7.1	-6.3
96	Emodin	3220	-7.1	-5.9
97	Chrysophanol	10208	-7.1	-6
98	Physcion	10639	-7.1	-5.9
99	Dehydrocavidine	92043552	-7	-6.5
100	Aloe-emodin	10207	-7	-6.3
101	Daucosterol	5742590	-7	-6.4
102	β-sitosterol	222284	-6.9	-6.7
103	Reticuline	439653	-6.9	-6.2
104	Resveratrol	445154	-6.9	-5.9
105	Oxyresveratrol	5281717	-6.9	-5.9
106	10-Hydroxyloganin	443340	-6.9	-5.5
107	Norjuziphine	15690955	-6.8	-6.2
108	Glycyrrhizin	14982	-6.8	-7.1
109	Dihydrosamidin	442128	-6.8	-6.5
110	Khayanthone	6708528	-6.8	-5.3
111	Valtratum	442436	-6.6	-5.8
112	Sennoside A	73111	-6.4	-6.1
113	Hibicuslide C	11276313	-6	-5.7
114	Hibiscus lactone	6481826	-6	-4.5
115	Scoparone	8417	-5.7	-5.2
116	Ferulic acid	445858	-5.7	-5.1
117	Larixol Acetate	4615087	-5.7	-6
118	Methyl gallate	7428	-5.6	-4.7
119	Caffeic acid	689043	-5.6	-5.2
120	N-acetylglucosamine-	24139	-5.6	-4.8
121	Ledene	10910653	-5.5	-5.4
122	2-Nonaprenyl-6-methoxyphenol	5372355	-5.4	-5.8
123	Bornyl acetate	6448	-5.4	-5
124	Protocatechuic acid	72	-5.4	-4.5
125	Hibiscus acid	123908	-5.3	-4.5
126	Methyl p-hydroxyphenyllactate	170975	-5.3	-4.9
127	Myristicin	4276	-5.3	-5.2
128	Linoleic acid	5280450	-5.2	-5.7
129	Caryophyllene oxide	1742210	-5.2	-6
130	Schizonepetin	86575555	-5.2	-5.3
131	β-caryophyllene	5281515	-5.2	-5.8
132	Germacrene D	5317570	-5.2	-5.7
133	Menthofuran	329983	-5.1	-5.1
134	L-ephedrine	9294	-5	-5.2

135	Terpinolene	11463	-4.9	-4.8
136	Piperitenone	381152	-4.9	-5.1
137	Pyrogallol	1057	-4.9	-4.3
138	p-Hydroxyacetophenone	7469	-4.8	-4.5
139	D-pseudoephedrine	7028	-4.8	-5
140	Pulegone	442495	-4.8	-5
141	Carvone	7439	-4.8	-5
142	β-Thujone	91456	-4.8	-5
143	Carvacrol	10364	-4.8	-5
144	Thymol	6989	-4.7	-4.9
145	Isopulegone	34645	-4.7	-4.7
146	Verbenone	29025	-4.7	-4.8
147	α-thujone	261491	-4.6	-4.8
148	O-cymene	10703	-4.6	-4.5
149	L-methylephedrin	4374	-4.6	-5.1
150	γ-Terpinene	7461	-4.6	-4.7
151	p-Cymene	7463	-4.6	-4.8
152	Alliin	87310	-4.5	-4.1
153	Carveol	7438	-4.5	-4.8
154	Camphor	2537	-4.4	-4.4
155	Limonene	22311	-4.4	-4.8
156	Stearic acid	5281	-4.4	-5.1
157	Sabinene	18818	-4.4	-4.7
158	Ajoene	5386591	-4.3	-4.1
159	Borneol	64685	-4.3	-4.6
160	α-pinene	6654	-4.3	-5
161	Palmitic acid	985	-4.3	-5
162	β-Myrcene	31253	-4.3	-5
163	Menthone	26447	-4.1	-4.1
164	Allicin	65036	-3.4	-3.8
165	Methyl allyl thiosulfinate	129712276	-3.4	-3.3
166	Allitridin	16315	-3.3	-3.6
167	β-propiolactone	2365	-3.2	-2.6
168	Garlicin	16590	-3.1	-3.4
169	Glycyrrhizate	62074	-1.3	-1.3
